# Understanding off-label use and the new challenges

**DOI:** 10.1186/1750-1172-9-S1-O22

**Published:** 2014-11-11

**Authors:** Marc Dooms

**Affiliations:** 1 University Hospitals Leuven, Belgium

## 

As there are several thousands of rare disorders and only some tents of orphan drugs authorized in Europe, off-label use of medicinal products occurs frequently in the diagnosis, prevention and treatment of rare diseases.

Off-label use is the use of a medicinal product for another indication, another patient group, another dose or by another route of administration as indicated in the package insert. This so-called off-label use is suggested by a similar mode of action or a similar pathology. The competent authorities complain about this therapeutic usage because there is no evidence of their safety and efficacy. Payers hesitate to reimburse this non-validated use. The only responsible person for this use is the prescriber. Sometimes the patient will be asked to sign an informed consent. Normalization of this off-label use can only be done by the sponsor not by the medical profession or the patient organization. Pharmaceutical companies are not allowed to mention the off-label use to the medical profession: recently settlements are reached against companies to resolve allegations of off-label promotion of their orphan pharmaceutical products (Tobi, Trisenox, Xyrem). Some pharmaceutical companies bring the old substance on the market as a so-called new “repurposed” or “rediscovered” medicinal product with a higher price (Litak, Quenobilan, Savene, Xenbilox). When the medicinal product is taken from the market (deflazacort, mexiletine) the off-label users lose their only treatment.

At the faculty of pharmacy of the University in Leuven, a group of students performed in-depth interviews to all the stakeholders in this process and identified the main obstacles to regularization. The Belgian Health Care Knowledge Center [https://kce.fgov.be/] will further explore these recommendations and give advice to the public authorities to take further actions. The Belgian Minister of Health already proposed a Royal Decree to reimburse some off-label use under restricted circumstances such as “unmet medical need”. EURORDIS has launched an off-label questionnaire within their members to explore the impact of this off-label prescribing for patients with rare diseases: http://www.ataxia.org.uk/news.php/234/off-label-use-of-medicines-information-needed-from-people-with-ataxia.

